# Effect of horizontal slot of maxillary canines’ brackets with varying wire angulations - An in vitro study

**DOI:** 10.1590/0103-6440202205104

**Published:** 2022-10-21

**Authors:** Márcio Crestana Cantarelli, Ana Paula Terossi de Godoi, Mário Alexandre Coelho Sinhoreti, José Guilherme Neves, Eduardo Almada Santos, Lourenço Correr-Sobrinho, Ana Rosa Costa

**Affiliations:** 1 Department of Orthodontics, Araras Dental School, Herminio Ometto Foundation, FHO, Araras, SP, Brazil; 2 Department of Restorative Dentistry, Dental Materials Division, Piracicaba Dental School, UNICAMP, State University of Campina, Piracicaba, SP, Brazil; 3 Department of Health, Orthodontics and Pediatric Dentistry, Piracicaba Dental School, UNICAMP State University of Campinas, Piracicaba, SP, Brazil

**Keywords:** Canines, Brackets, Dental movement techniques

## Abstract

A new device was developed to enable the visualization and measurement of canine angulation while at the same time visualizing and measuring the force transmitted to adjacent teeth. This study aimed to evaluate the mesiodistal tilt angle of the upper canine brackets, the wire deflection, and its effects on adjacent teeth with five different slot designs of upper canines. Wires (0.020” and 0.019” x 0.025”) were tested on different five bracket types at five different distal angles. The force applied to adjacent teeth was measured as the angle was increased, and its consequences were observed in the posterior and anterior regions as well. The force tension (gf) was measured in a universal testing machine. Data were submitted to a 3-way ANOVA and Tukey’s test (α=0.05). For both arches, regardless of the type of tooth and bracket type, the highest means tension mean values were shown by the 20° angle, followed by the 15°,10°, and 5° angles, which differed statistically among themselves. Overall, for 5°, 10°, and 15° angles, conventional and versatile brackets showed significantly higher force values in all teeth, tip-edge and control brackets showed the lowest. The highest force values were observed in central and lateral incisors with conventional and versatile brackets and on first and second premolar teeth with self-ligating passive and control brackets. Conventional brackets presented the highest forces, tip-edge and control brackets showed the lowest. The teeth that suffered the greatest forces were lateral incisors, and those that suffered the least were second premolars.

## Resumo

Foi desenvolvido um novo dispositivo para permitir a visualização e medição da angulação canina ao mesmo tempo que se visualiza e mede a força transmitida aos dentes adjacentes. Este estudo avaliou o ângulo de inclinação mesiodistal dos braquetes caninos superiores, a deflexão do fio, e os seus efeitos nos dentes adjacentes com cinco diferentes desenhos de ranhuras dos caninos superiores. Os fios (0,020" e 0,019" x 0,025") foram testados em cinco diferentes tipos de braquetes e ângulos distais. A força aplicada aos dentes adjacentes foi medida a medida que o ângulo era aumentado, e as suas consequências foram observadas também nas regiões posterior e anterior. A tensão da força (gf) foi medida em uma máquina de ensaio universal (Instron). Os dados foram submetidos ao teste ANOVA 3 fatores e Tukey (α=0,05). Para ambos os arcos, independentemente do tipo de dente e tipo de braquete, os valores médios de tensão mais elevados foram mostrados pelo ângulo de 20°, seguido pelos ângulos de 15°, 10°, e 5°, que diferiram estatisticamente entre si. Em geral, para ângulos de 5°, 10° e 15°, os braquetes convencionais e versáteis apresentaram valores de força significativamente mais elevados em todos os dentes, braquetes tip-edge e controle apresentaram os mais baixos. Os maiores valores de força foram observados nos incisivos centrais e laterais com braquetes convencionais e versáteis e no primeiro e segundo pré-molares com braquetes auto-ligantes passivo e controle. Os braquetes convencionais apresentaram as forças mais elevadas, os braquetes controle e tip-edge apresentaram os valores mais baixos. Os dentes que sofreram as maiores forças foram os incisivos laterais, e os que sofreram menos foram os segundos pré-molares.

## Introduction

Due to the movement of dental verticalization, side effects are observed arising from the prescription of final mesial inclination, which is embedded into horizontal channels of canine brackets. This prescription can deform the orthodontic wire, which exerts an action to correct the position of a particular tooth and a reaction in adjacent teeth ^1^.

Focusing on the upper canines, if a distal movement is necessary, which is common, a sequence of inclinations and verticalization will begin. When tilting it towards the distal, its horizontal channel tilts as well, and detailing that inclination, the anterior part of the horizontal channel faces the incisal of the anterior teeth. This tilting promotes anterior extrusive movement, while the posterior part of the horizontal channel faces the gingival of the posterior teeth, promoting an intrusive movement in the premolar region.

With such a prescription, either in the alignment and leveling phase or in the retraction and extraction spaces closure phases, side effects caused by the passage of the wire, besides its caliber through this channel, cause an anterior deep bite and an open bite at the premolar region due to wire deflection ^2^. This occurs by wire bending caused by its contact with the edges of the horizontal channel when tilted distally. Several solutions were adopted to avoid these side effects, one of which was to isolate the canine from the continuous wire, as the canine would be the cause of these effects ^3^.

To reduce these adverse effects and overcome the complications of dental movement, some methods have been used in addition to redesigning the horizontal channel walls ^4^ ^,^ ^5^ ^,^ ^6^ ^,^ ^7^ ^,^ ^8^. To counteract these unwanted side effects, Andrews ^9^ incorporated into these brackets' design inverse characteristics to the movements they would tend to make during translation, such as anti-angulation.

Thus, the present study aimed to evaluate the mesiodistal inclination angulation of upper canine brackets, the wire deflection, and its effect on the adjacent teeth of five brackets with different models of horizontal channels when inclined distally. The null hypotheses of the present study were that the wire deflection (gf) would be 1) similar for the different angles of 5º, 10º, 15º, and 20º; 2) the same for different bracket designs; and 3) similar in the adjacent teeth (central incisor, lateral, first and second premolars).

## Material and methods

### Brackets and Steel Wires

**Figure 1:**
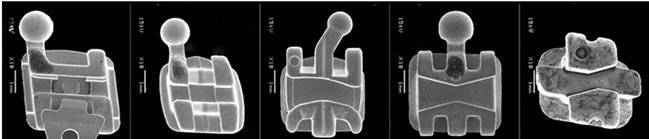
Brackets morphology on scanning electronic microscopy (SEM). From left to right: Self-ligating passive Bracket; Conventional straight-wire bracket; Versatile bracket; Control bracket; and, Tip-edge bracket.

Five types of commercial right upper canine brackets (Rooth prescription and 0.022” x 0.028” slots - Figure 1) were used in this study - self-ligating passive (Morelli, Sorocaba, São Paulo, Brazil); conventional straight-wire (Morelli); versatile (Dentsply Sirona GAC International, Iceland, NY, USA); control (Morelli,); and tip-edge (TP Orthodontics, La Porte, Indiana). Steel wires (Morelli) with different diameters (0.020” and 0.019” x 0.025”) were attached to the brackets by an elastic ligature (Morelli)*.*

### Development of Device

**Figure 2:**
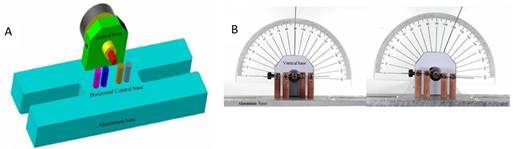
A. 3D drawing of the innovative device developed for the present study. Pink vertical celeron piston represents the second premolar; Blue - first premolar; Yellow - lateral incisor; Gray - central incisor; and, Red - horizontal celeron piston represents canine. B. Device photography was developed to measure and visualize canine inclination.

The brackets were mounted on a specific device developed (Figures 2A and B), which enabled visualization and measurement of canine angulation while at the same time visualizing and measuring the force transmitted to adjacent teeth. The device was designed and manufactured on an aluminum base, channels were drilled in the central part of the device to be attached to a universal testing machine (Instron; 4411 model, Canton, MA, USA). Celeron pistons were inserted into four vertical channels that simulated the intrusion and extrusion movement of adjacent teeth to the canine. To reproduce the mesiodistal inclination of canines, a perpendicular bulkhead was fixed to the horizontal base, with a hole where a handle was inserted with a socket for a cylinder in a horizontal position that received a canine bracket. The brackets from each prescription were fixed on vertical pistons, where each piston received the right upper brackets (central and lateral incisors, first and second premolars) (Figure 2B). On the vertical base, a protractor and a metal rod were attached to indicate the angle as the canine was tilted (Figures 2A and B).

### Tension (gf)

Wire replacement, bracket prescriptions, piston positioning, canine angulation, and device locking were performed by a single operator. The steel wire was passively installed in the device, and tension (gf) was measured at 4 angles (5º, 10º 15º, and 20º) when the canine arrived at the desired angulation on Instron, the system was locked in this situation, which recorded the information over a period of 20 seconds, recording maximum, minimum, and average tension of each situation and exporting data to an Excel spreadsheet. The wire was changed at each angle.

### Statistical Analysis

The tension data (gf) were submitted to a 3-way ANOVA (angulation x bracket x tooth) and Tukey´s test for multiple comparisons (α = 0.05) (SAS User´s Guide: Statistics, version 9.4. Cary [NY]: SAS Institute Inc 2001).

## Results

The mean (standard deviation) of tension (gf) after deflection of round wires can be seen in Table 1. Regardless of tooth type and bracket design, the 20° angle showed the highest tension mean values, followed by the 15°, 10°, and 5° angulation, all of which differed statistically from each other. For 5° angle, in general, tip-edge, self-ligating passive, and versatile brackets showed the lowest tension mean values significantly in all teeth, and the conventional showed the highest rates. The highest tension mean values were observed in the second premolar and lateral incisor teeth and the lowest in the central and first premolar teeth.

**Table 1:** Mean (standard deviation) of tension (gf) after deflection of orthodontic round wires (0.020”) inserted in different designs of orthodontic brackets according to tooth type and deflection angulation.

Angulation*		Central	Lateral	1^st^ Premolar	2^nd^ Premolar
	Design				
5 ^o^ 25.05 (5.36) d	Conventional	90.52 (6.62) a,B	106.37 (4.96) a,A	20.42 (1.43) b,C	28.00 (0.12) b,C
	Versátil	0.93 (0.11) c,B	0.49 (0.07) c,B	6.13 (0.42) cd,B	17.02 (1.31) c,A
	SLP	0.82 (0.00) c,C	0.52 (0.11) c,C	44.96 (2.20) a,B	55.35 (0.95) a,A
	Control	47.68 (1.16) b,B	56.47 (4.53) b,A	1.88 (0.72) d,C	0.90 (0.07) d,C
	Tip-Edge	0.19 (0.07) c,B	0.27 (0.00) c,B	11.63 (0.31) c,A	10.48 (0.67) c,A
10 ^o^ 92.47 (7.77) c	Conventional	266.89 (5.77) a,C	333.09 (5.98) a,A	280.78 (11.88) a,B	51.32 (3.31) c,D
	Versátil	155.84 (5.92) b,B	211.60 (5.75) b,A	40.43 (0.83) d,D	64.08 (2.04) b,C
	SLP	35.72 (0.39) c,D	86.91 (1.17) c,B	114.37 (3.51) b,A	73.71 (1.23) a,C
	Control	25.65 (5.17) d,A	25.89 (4.05) d,A	0.71 (0.05) e,B	0.55 (0.00) d,B
	Tip-Edge	9.81 (0.08) e,B	0.14 (0.00) e,C	69.99 (1.02) c,A	2.00 (0.48) d,BC
15 ^o^ 129.63 (15.8) b	Conventional	253.65 (7.06) b,B	429.67 (6.13) a,A	199.58 (13.87) b,C	60.96 (1.22) b,D
	Versátil	306.75 (2.49) a,B	325.45 (4.14) b,A	145.36 (7.56) c,C	36.10 (5.02) c,D
	SLP	150.94 (2.90) c,B	82.37 (1.61) c,D	216.01 (7.03) a,A	119.18 (2.13) a,C
	Control	41.25 (2.43) d,B	51.02 (2.59) d,A	49.76 (2.74) d,A	12.40 (0.41) d,C
	Tip-Edge	0.27 (0.00) e,B	0.14 (0.00) e,B	56.85 (0.85) d,A	56.06 (4.01) b,A
20 ^o^ 205.52 (16.0) a	Conventional	270.18 (11.64) b,C	715.18 (5.48) b,A	352.25 (16.49) a,B	115.76 (0.40) a,D
	Versátil	400.95 (10.62) a,B	837.77 (14.79) a,A	303.06 (1.69) b,C	21.84 (0.43) c,D
	SLP	160.93 (1.08) c,C	329.39 (3.05) c,A	310.15 (2.68) b,B	120.96 (1.35) a,D
	Control	63.31 (7.99) d,A	2.13 (0.83) d,C	28.27 (6.74) c,B	23.35 (0.20) c,B
	Tip-Edge	2.87 (0.21) e,C	0.29 (0.06) d,C	17.76 (4.90) d,B	34.08 (0.34) b,A

Different uppercase letters in line and lowercase letters in column indicate statistically significant difference at *P* < .0001. * Comparison of spine angulation means, regardless of bracket design and tooth type. *3-way* ANOVA showed that the factors "bracket" (*P* = .00001), "tooth" (*P* = .00001) and "angulation" (*P* = .00001) were significant. The double interaction between the factors "bracket x tooth" (*p* = .00001), "bracket x angulation" (*P* = .00001) and "tooth x angulation" (*P* = .00001) were also significant, as well as the triple interaction "bracket x tooth x angulation" (*P* = .00001).

In the 10° and 15° angles, in general, tip-edge and control brackets showed the lowest tension mean values significantly in all teeth. The conventional bracket showed the highest, except in the second premolar, where the self-ligated bracket showed the highest tension averages (10°). The highest values were observed with the conventional and versatile brackets in the central and lateral incisor teeth and the self-ligating passive bracket in the two premolar teeth (15°). Considering the teeth types, the highest tension mean values were observed in the first premolar and lateral incisor teeth and the lowest in the second premolar, except when the tip-edge bracket was used (15°).

 Generally speaking, at the 20° angle, the tip-edge and the control brackets showed significantly the lowest tension mean values in all teeth except the second premolar, where the versatile and the control brackets showed the lowest. The highest values were observed with the conventional and versatile brackets in all teeth except the second premolar, where the conventional and self-ligating passive brackets presented the highest. Comparing tooth types, the highest tension mean values were observed in the lateral incisor tooth, except when tip-edge (second premolar) and control brackets (central incisor) were used. The lowest tension was observed in the second premolar tooth, except when the tip-edge bracket was used.

Table 2 shows the mean (standard deviation) of tension (gf) after the deflection of rectangular wires. Regardless of tooth type and bracket design, the 20° angle showed the highest tension mean values, followed by the 15°, 10°, and 5° angulation, all of the wich differed statistically from each other. For 5° angulation, in general, the conventional and versatile brackets showed significantly the highest tension mean values ​​in all teeth, and the tip-edge and control brackets showed the lowest. The highest tension mean values ​​were observed in central and lateral incisor teeth for the conventional and versatile brackets and in the first premolar and second premolar teeth for the self-ligating passive and control brackets.

**Table 2:** Mean (standard deviation) of tension (gf) after deflection of orthodontic rectangular wires (0.019” x 0.025”) inserted in different designs of orthodontic brackets according to tooth type and angulation of the deflection.

Angulation *		Central	Lateral	1^st^ Premolar	2^nd^ Premolar
	Design				
5 ^o^60.0 (3.25) d	Conventional	275.82 (5.92) a,B	382.83 (13.80) a,A	12.86 (5.92) b,D	32.60 (0.29) b,C
	Versátil	104.92 (12.05) b,A	102.13 (1.55) b,A	20.24 (9.83) b,B	17.13 (3.84) bc,B
	SLP	29.37 (4.07) c,B	37.31 (1.80) c,B	57.04 (6.45) a,A	27.18 (0.55) b,B
	Control	0.98 (0.14) d,B	0.03 (0.02) d,B	23.68 (11.12) b,A	1.56 (0.94) c,B
	Tip-Edge	-0.87 (0.07) d,C	-0.76 (0.07) d,C	24.63 (2.84) b,B	51.43 (1.55) a,A
10 ^o^157.6 (10.53) c	Conventional	427.48 (8.27) a,A	339.09 (27.32) b,B	117.08 (14.81) c,C	121.73 (4.96) a,C
	Versátil	435.48 (15.76) a,A	389.48 (2.66) a,B	295.37 (32.27) a,C	122.50 (6.65) a,D
	SLP	230.27 (5.27) b,B	196.41 (9.57) c,C	256.36 (8.92) b,A	98.02 (5.21) b,D
	Control	8.54 (1.64) c,B	-0.32 (0.07) d,B	7.52 (3.65) e,B	35.77 (2.32) c,A
	Tip-Edge	-1.09 (0.00) c,B	-0.95 (0.01) d,B	61.04 (1.45) d,A	13.11 (2.12) d,B
15 ^o^ 229.1 (21.27) b	Conventional	434.59 (13.96) b,A	209.52 (29.43) c,C	335.55 (25.52) c,B	125.32 (5.56) b,D
	Versátil	455.37 (21.54) a,A	846.97 (17.12) a,B	595.06 (9.96) a,C	208.78 (4.93) a,D
	SLP	352.53 (1.22) c,B	289.07 (5.63) b,C	428.90 (3.93) b,A	198.33 (0.94) a,D
	Control	3.28 (0.78) d,B	0.00 (0.00) d,B	24.00 (11.06) d,A	9.99 (0.91) d,AB
	Tip-Edge	-0.98 (0.01) d,B	-1.06 (0.04) d,B	24.80 (3.61) d,A	41.99 (4.11) c,A
20 ^o^ 329.9 (23.94) a	Conventional	284.91 (11.16) c,B	274.56 (24.69) c,B	398.71 (23.20) c,A	234.84 (1.24) c,C
	Versátil	567.42 (22.32) a,C	936.89 (22.27) a,B	1,052.68 (18.52) a,A	347.24 (3.71) a,D
	SLP	375.52 (8.53) b,C	636.02 (5.09) b,A	575.68 (1.72) b,B	300.76 (8.78) b,D
	Control	97.97 (9.00) d,C	181.60 (7.45) d,A	133.26 (10.31) d,B	87.35 (3.00) d,C
	Tip-Edge	-1.08 (0.01) e,B	-0.98 (0.05) e,B	58.47 (0.99) e,A	57.75 (1.15) e,A

Different uppercase letters in line and lowercase letters in column indicate statistically significant difference at *P* < .0001. * Comparison of spine angulation means, regardless of bracket design and tooth type. *3-way* ANOVA showed that the factors "bracket" (*P* = .00001), "tooth" (*P* = .00001) and "angulation" (*P* = .00001) were significant. The double interaction between the factors "bracket x tooth" (*p* = .00001), "bracket x angulation" (*P* = .00001) and "tooth x angulation" (*P* = .00001) were also significant, as well as the triple interaction "bracket x tooth x angulation" (*P* = .00001).

At the 10° angle, also, in general, tip-edge and control brackets showed significantly the lowest tension mean values in all teeth, and versatile and conventional brackets showed the highest, except in the first premolar tooth, where self-ligating and versatile brackets showed the highest tension averages. Considering tooth types, the highest tension mean values were observed in central and lateral incisors with the versatile and conventional brackets and in the first and second premolars when the control and tip-edge brackets were used.

For the 15° angle, in general, tip-edge and control brackets showed significantly the lowest tension mean values in all teeth. The highest values were observed with the versatile bracket, also in all teeth. Considering teeth types, the highest tension mean values were observed in the central incisor tooth with the versatile and conventional brackets and in the first and second premolars when the control and tip-edge brackets were used.

Generally considering the 20° angle, the tip-edge bracket showed significantly the lowest tension mean values in all teeth, followed by the control, conventional, self-ligating passive, and versatile brackets, which differed statistically from each other. Comparing tooth types, the highest tension mean values were observed in the first premolar with the versatile, conventional, and tip-edge brackets and in the lateral incisor when the control and self-ligating passive brackets were used. The lowest tension was observed in the second premolar, except when the tip-edge bracket was used.

**Figure 3:**
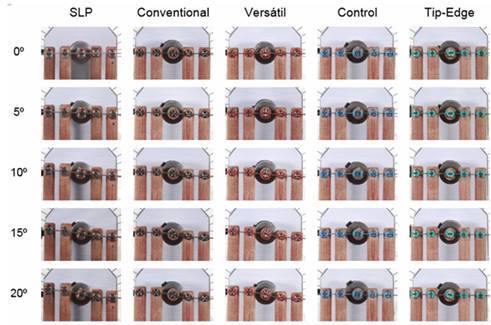
Behavior of the round steel wire (0.020”) at different angles of the canines. At 0º, all prescriptions had no deflection of the wire (passive system) . Slight wire deflection was observed in some prescriptions (5º). At 10º and 15º the deflection increased as the degree of canine inclination increased. At the highest inclination (20º), tip-edge and control brackets transferred the least force, and conventional and versatile brackets transferred the most.

**Figure 4:**
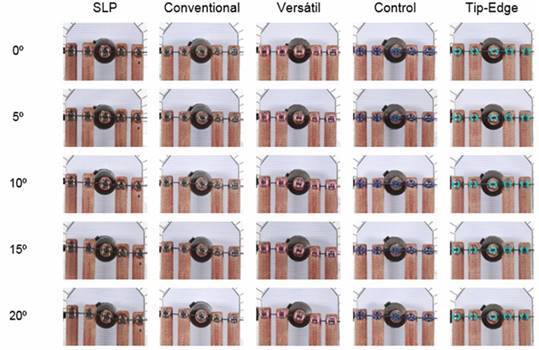
Rectangular steel wire (0.019”x0.025”) behavior at different canine angles. At 0º, there was a passive system in all prescriptions. Thus, as the canine is tilted distally, the deflection of the wire is observed and thus its effect to adjacent teeth (5º). At 10º and 15º, a tendency to extrude anterior teeth and intrude at the premolar region is clearly seen in the first three columns, and how rectangular wire remains unchanged in its rectilinear form in the last two. At the highest angle (20º), control and tip-edge brackets transferred the least force, while versatile and conventional brackets transferred the most.

The behavior of both orthodontic wires in different canine angles is shown in Figures 3 and 4, respectively.

## Discussion

The first hypothesis of the present study was rejected, because the deflection of round or rectangular wire, regardless of teeth and bracket type, was different for different angles. The results of the present study showed that the 20° angulation presented the highest deflection mean values, followed by the 15°, 10°, and 5° angles, which differed statistically from each other. The results of the present study corroborate those of a previous study ^10^. As the angular contact relationship between the bracket channel and the wire increases or decreases depending on how much the tooth tilts or verticalizes during movement ^10^ adverse situations occur just to reach the final mesiodistal inclination prescriptions.

The wire deflection that is observed in cases of misaligned teeth occurs when the gap between the arch and the horizontal channel disappears, producing a binding effect. Therefore, even a slight misalignment between the wire and the horizontal channel would be enough to trigger the process ^11^.

This effect may delay tooth movement in the active unit, while the reactive unit begins to move, causing anchorage loss ^12^. When the line of action of this force moves below the center of resistance of the anterior teeth, a backward force moment affects the anterior teeth, resulting in incisor inclination and extrusion ^12^. The forces that resist the sliding motion are frictional forces and arch deflection ^13^. Although too much attention is given to frictional force, the forces caused by arch deflection would be greater, making the tooth movement difficult ^13^. In addition, among the characteristics that influence canine distalization mechanics, attention should be given to the size of bracket slots and the wire thickness ^14^ ^,^ ^15^ ^,^ ^16^ ^,^ ^17^.

Body movement does not occur by itself; is a series of movements between the horizontal channel width and the arch thickness difference, allowing the tooth to tilt with subsequent arch deflection promoted by the horizontal channel. The overall result of this deflection is noted by the bite deepening and/or the increased force level required to prevent this effect, except with tip-edge and control brackets. Brackets that have wedges removed from their horizontal channels allow the arch not to deflect at the moment the canine tilts to the distal, which does not occur in the conventional brackets because they have straight horizontal channels which exacerbate the channel's contact angle with the wire, deflecting it and causing the bite to open in the premolar region and deepening in the anterior region. The versatile bracket has a mesial extension in its horizontal channel and does not allow such distal freedom, resulting in an anterior deep bite and a lateral open bite and/or in an increase in the required force level with increased angulation in the rectangular wire from 104.92 gf (5º) to 567.92 gf (20º) and the round wire from 0.93 gf (5º) to 400.93 gf (20º).

The second hypothesis of this study was also rejected. The deflection of the round or rectangular wire (gf) was not the same with the different bracket models. The inclination of the different horizontal channel designs and the force transmission were significantly different for the different bracket types. In general, for the round and rectangular steel wires, the highest tensions transmitted in relation to the angle were observed with the conventional, self-ligating passive and versatile brackets, and the lowest tensions by tip-edge and control brackets, for both wires. The horizontal channel design of straight-wire brackets could cause difficulties at the beginning of the treatment that might be difficult to correct ^2^ ^,^ ^3^ ^,^ ^7^ ^,^ ^17^.

The inclination prescription that is incorporated into canine brackets is the main problem when an arch inserted into a verticalized or distal inclined canine bracket can promote incisor extrusion ^2^ ^,^ ^7^ ^,^ ^10^ ^,^ ^17^. In addition, the natural tendency of the canine crown to tilt distally during retraction increases the extrusive effect ^2^. Using conventional pre-adjusted brackets, the deleterious effects caused by the distal inclination of the upper canines at the anterior retraction and even in the early stages, when canine verticalization is present, are clinically observed ^2^ ^,^ ^7^ ^,^ ^17^.

The undesirable effects on adjacent teeth were statistically different for different types of brackets and wires, resulting in the rejection of the third hypothesis as well. For round wire, the teeth that presented the greatest adverse effects were the lateral incisor followed by the first premolar, second premolar, and central incisor. The lowest values were observed in the second premolars, followed by the first premolars, central, and lateral incisors. For rectangular wire, the teeth that suffered the greatest adverse effects were the first premolar followed by the central, lateral incisors and second premolar. Few or no studies have been found in the literature indicating which teeth would be directly and/or indirectly affected by the magnitude of posterior intrusion and anterior extrusion movements caused by round or rectangular wire deflection due to the different designs used in canine brackets.

Andrews ^9^ noted that the dental elements most affected by translation movement were the canines, premolars, and molars, because they exhibited unwanted movements during translation, such as rotations and angulations of their crowns. To reduce adverse effects caused by wire deflection, brackets that have horizontal channels designed to avoid these problems can be used. Based on conventional edgewise brackets, the horizontal channel design of tip-edge and control brackets are characterized by the removal of opposite diagonal corners on the horizontal channel to allow for greater mesial or distal inclination of the teeth ^2^ ^,^ ^3^ ^,^ ^6^ ^,^ ^17^ according to the results of the present study.

 During tilt movements, due to sliding mechanics, channels that are 0.022” in size can reach up to 0.028” in tip-edge brackets only, due to the removal of wedges, which are not diametrically opposed, and to a lesser extent in control brackets (Figure 1 - diametrically opposed wedges), reducing frictional force and arch deflection, facilitating orthodontic mechanics, and configuring a passive arch-wire situation ^3^ ^,^ ^10^ ^,^ ^16^. Due to the horizontal channel design of this bracket, when a tooth is inclined by the retraction movement, the binding effect is minimized and frictional resistance is reduced ^10^ ^,^ ^15^ ^,^ ^16^. The results of the present study corroborate with those of Kesling ^6^, who observed that the horizontal channel of the tip-edge bracket enabled the closure of the extraction spaces or the retraction of the entire arch by tilt and subsequent verticalization with a small or no vertical arch deflection. The same situation was observed for the control bracket in the present study.

Despite all these positive characteristics of the brackets that allow a distal inclination without wire deflection, we should not ignore the positive characteristics of other brackets. For those treatments for which the distal tilt of the crown is needed, control and tip-edge brackets are indicated, and for the others for which the opposite characteristics are needed as well, the self-ligating, conventional and versatile brackets could be used. What must be emphasized is how much tension forces affect the adjacent teeth according to the bracket prescription adopted.

Thus, the combination of conventional straight-wire brackets with brackets that have a different canine horizontal channel design, such as tip-edge and control, avoids adverse situations such as wire deflection, anterior bite deepening, bite opening at the premolar region, and transmission of unwanted forces to adjacent teeth, facilitating distalization movements of the anterior battery.

The present study has brought another tool for the Orthodontist who, in his clinical practice, is commonly found, with the adverse situation of deepening of bite in the anterior battery and opening in the region of premolars in the retraction phases, distalization of the upper canines and even in the initial stages of food and leveling, when using conventional straight-wire brackets.

It is worth mentioning, that lighter wires are used in the initial phases for alignment and leveling where the inclination of the canines may be present. However, the intention was to show that even with arches of a greater caliber the inclination of the canine happens in the retraction and distalization of the canine. It is notorious that with the clinical experience situations such as opening the bite in the region of premolars and deepening of the previous bite are adverse effects that happen even with heavy wires. Changing brackets of conventional canines by modified channels might reduce or inhibit these adverse effects. And the fact of provoking exaggerated situations was to show that not even then adverse effects are avoided.

## Conclusion

1. . The relationship between arch and inclination of the horizontal channel significantly affected the arch deflection;
2. . Control and tip-edge brackets allowed the distalization and mesialization of canines without deflecting arches, configuring a passive situation;
3. . Brackets that have conventional horizontal channels (self-ligating passive and conventional straight-wire) directly affected wire deflection as the teeth tilted distally;
4. . Versatile bracket, due to the mesial extension of its horizontal channel, did not allow such distal freedom; and,
5. . Lateral incisors and first premolars were the teeth that suffered the greatest load due to the wire deflection.
